# Development of a cheminformatics platform for selectivity analyses of carbonic anhydrase inhibitors

**DOI:** 10.1080/14756366.2019.1705291

**Published:** 2019-12-19

**Authors:** Giulio Poli, Salvatore Galati, Adriano Martinelli, Claudiu T. Supuran, Tiziano Tuccinardi

**Affiliations:** a Department of Pharmacy, University of Pisa, Pisa, Italy; b NEUROFARBA Department, Sezione di Scienze Farmaceutiche, Università degli Studi di Firenze, Florence, Italy

**Keywords:** Carbonic anhydrase, fingerprint, clustering, selectivity

## Abstract

The selectivity for a specific human Carbonic Anhydrase (hCA) isoform is an important property a hCA inhibitor (CAI) should be endowed with, in order to constitute a valuable therapeutic tool for the treatment of a desired pathology. In this context, we developed a chemoinformatic platform that allows the analysis of the structure and selectivity profile of known CAIs reported in literature, with the aim of identifying structural motifs connected to ligand selectivity, thus providing useful guidelines for the design of novel ligands selective for the desired hCA isoform. The platform is able to perform ultrafast structure and selectivity analyses through ligand fingerprint similarity, with no need of structural information about the target receptor and ligands’ binding mode. It is easily accessible to the non-expert user through the implementation of a KNIME Analytic Platform workflow and could be extended to analyze the selectivity profile of known ligands of different target proteins.

## Introduction

Human Carbonic Anhydrases (CAs) constitute a superfamily of metalloenzymes characterized by the presence of a zinc (Zn^2+^) ion as prosthetic group, which is necessary for their catalytic activity. At present, seven different genetic families of CAs have been widely acknowledged: α-, β-, γ-, δ-, ζ-, η- and θ-CAs. All these enzymes catalyze the zinc-mediated reversible hydration of CO_2_ in HCO_3_^–^ and H^+^, although some secondary catalytic reactions have been discovered and reported for at least some classes of CAs^1^. Among the six genetic CA families, α-CAs are certainly the most studies enzymes, since they are expressed in humans as in other mammalians and vertebrates; in particular, 16 different human CA isoforms have been identified to date: hCA I–IV, hCA Va, hCA Vb and hCA VI–XV^1^. These isoforms are characterized by different tissue and subcellular localization and are therefore implied in the plethora of physiological processes in which the synthesis, transport and homeostasis of carbon dioxide play an important role. For instance, hCAs are involved in intracellular pH regulation, electrolytes secretion, gluconeogenesis, lipogenesis and bone resorption. It is thus easy to believe that hCAs are also connected to the development and progression of numerous pathologies, such as obesity, epilepsy, glaucoma, osteoporosis and cancer^2^. Therefore, small-molecules endowed with inhibitory activity towards specific hCA isoforms can be used as therapeutic tools for the treatment of these diseases. In this context, the selectivity of CA inhibitors (CAIs) for specific hCA isoform is crucial for obtaining the desired therapeutic effect. hCA II is the most studied hCA isoform due to its localization in most tissues and organs. Together with other CA isoforms, hCA II is targeted by clinically used drugs such as acetazolamide, methazolamide and dichlorophenamide, for the systemic treatment of glaucoma^3^. However, the widespread distribution of hCA II makes this isoform a common off-target of CAIs designed to interact with different hCA isoforms. As an example of the potential therapeutic role of the other CAs, the mitochondrial isoform hCA Va is considered a potential target for the treatment of obesity, diabetes and related disease, and various CAIs with selectivity for this isoform have been designed^4^. hCA VII is predominantly present in the central nervous system and thus selective hCA VII inhibitors can be used for the modulation and treatment of neuropathic pain^5^^,^^6^. hCA IX was found to be overexpressed in many different solid tumors and to be involved in the development of metastasis, thus selective hCA IX inhibitors represent promising anticancer agents^7^^,^^8^. Due to the many different pathological implications they are involved with and their therapeutic importance, hCAs constitute a well-studied family of drug targets within the medicinal chemistry field. This is proved by the remarkable volume of scientific literature focused on hCAs, counting almost fifteen thousand of research and review articles. Nevertheless, the interest on hCAs does not appear to be in decline. On the contrary, the recent studies confirming the potential of hCA IX and hCA XII as valuable targets for the treatment of hypoxic tumors spread a renewed interest in the identification of new and selective CAIs^9–13^. From one side, the availability of a large amount of literature data can surely facilitate the design of novel compounds with both high activity and selectivity for the desired hCA isoforms. On the other hand, an efficient way to explore such a wide ensemble of data appears to be necessary to avoid being overwhelmed by facts and figures, eventually losing the key information we are actually looking for. For this purpose, we aimed at developing a chemoinformatic platform that could allow a rapid and systematic analysis of the publicly available structural and biological data relative to CAIs, able to analyze information about ligand selectivity toward different hCA isoforms and to derive useful structure-activity relationships for the design of novel CAIs with selectivity for the desired target hCAs.

## Materials and methods

### Data collection and preparation

All data related to known hCA inhibitors were retrieved from PubChem database^14^; data download and processing were performed using in-house python scripts. Thirteen different hCA isoforms were taken into account in this study, namely hCA I–IV, Va, Vb, VI, VII, IX and XI–XIV. For each hCA isoform, PubChem website was used to download the list of compounds for which biological activity data for the corresponding enzyme were stored in the database, thus obtaining thirteen different lists of ligand records. No bioactivity data relative to small-molecules tested for hCA VIII, X and XV inhibition were instead found. In each downloaded list, each ligand record included the corresponding PubChem compound identification code (CID) and assay identification code (AID), the type of biological assay performed on the ligand and the corresponding experimental activity value expressed as either *K*_i_, IC_50_, *K*_d_, *K*_m_ or EC_50_, depending on the assay type. For our analyses, only the ligands associated to a *K*_i_ value for the corresponding hCA isoform were taken into account, while the other ones were filtered out. Moreover, the compounds were further filtered by retrieving the PubChem ID code (PMID) associated with their AID and selecting only ligands whose *K*_i_ values were determined in the laboratory of Prof. Claudiu T. Supuran in order to minimize the biases associated to the application of different experimental procedures. Subsequently, for those ligands presenting more than one *K*_i_ value (derived from different experimental assays) for the corresponding hCA, the different activity values were averaged prior removal of all potential outliers, thus obtaining a reliable mean *K*_i_ value. Finally, the structures of all ligands in SMILES notation were automatically downloaded from PubChem database. The thirteen refined datasets of ligands were then merged into a single spreadsheet containing the records of all compounds presenting a *K*_i_ value for at least one out of the 13 hCA isoforms considered in this study. Each ligand record included the CID code and the SMILES string of the compound, together with at least a *K*_i_ value. The full list of CAIs was then imported into Instant JChem^15^ software and subjected to a substructure filter in order to select only compounds bearing a sulfonamide fragment as zinc binding group (ZBG). With the application of this last filter, the final Instant JChem database of hCA inhibitors, including a total of 2837 ligands, was eventually obtained to be used for selectivity profile analyses.

### Preliminary data analysis

For the hCA IX/hCA II selectivity profile analysis, Instant JChem was used to select a subset of ligands showing *K*_i_ values for both hCA II and IX, corresponding to 1756 compounds. The software was also used to calculate the selectivity index (SI) of each ligand, defined as the ratio between hCA II and hCA IX *K*_i_ values. Based on this definition, a ligand with a 10-fold selectivity for hCA IX over hCA II would show a SI = 10. Analogously, Instant JChem was used to select the 1388 ligands with *K*_i_ values for both hCA II and XII and to calculate their SI values (ratio between hCA II and hCA XII *K*_i_ values) to be used for the hCA XII/hCA II selectivity profile analysis, as well as to select the 1322 compounds with *K*_i_ values for hCA II, IX and XII employed in the double selectivity analysis.

### Fingerprint analyses

The generation of ligand fingerprints and the calculation of the Tanimoto similarity index were performed using the pybel module of OpenBabel software^16^. All fingerprints were generated starting from the SMILES strings of the analyzed ligands. Four different fingerprint types available in OpenBabel were used: FP2, FP3, FP4 and MACCS fingerprints. FP2 is a path-based fingerprint that indexes ligand fragments based on linear segments of 1–7 atoms, while FP3, FP4 and MACCS fingerprints are based on different sets of SMARTS patterns, which are used to index ligand fragments. The Tanimoto similarity index (Ti) was calculated between pairs of ligand fingerprints as previously reported^17^, based on the following equation:

$$\mathrm{Ti}\;=\frac{\left|A\cap B\right|}{\left|A\cup B\right|}$$

where A∩B is the number of switched-on bits common to the fingerprint strings A and B, while A∪B is the sum of them. For each of the three previously selected subsets of ligands (with the SI values for hCA II/IX, hCA II/XII and hCA II/IX/XII), in-house python scripts were used to generate two different N × N matrices containing two different parameters calculated for all possible couples of elements included in the dataset: (a) the Ti of all possible pairs of ligand fingerprints and (b) the corresponding Tanimoto distance (Td, defined as 1 – Ti) of each fingerprint pair. When analyzing the subset of ligands with SI for hCA II/IX, the selectivity ratio (SR), defined as the ratio between the SI of two ligands expressed in logarithmic scale, was also calculated for all the corresponding pairs of compounds.

### Hierarchical clustering and cluster analysis

The clustering analysis was performed using the software orange-canvas^18^ based on FP2 and MACCS ligand fingerprints. For each of the three selected subsets of ligands, the compounds were clustered based on their reciprocal structural similarity, described by the previously calculated matrix of Td values. The single-linkage method was used as clustering algorithm. This method is an agglomerative type of hierarchical clustering that starts considering each element in a cluster of its own^19–21^. The clusters are then sequentially combined into larger ones, until all elements are in the same cluster. At each step, the two clusters separated by the shortest distance, corresponding to the distance of the most similar members of the clusters, are combined. For this analysis, the clusters were generated using a balanced distance cut-off, setting a 30% height ratio in the full clustering dendrogram; in this way, the generated clusters included groups of ligands with maximum reciprocal Td around 0.2 (and thus maximum reciprocal Ti around 0.8) when FP2 fingerprints were used, while a Td cut-off around 0.1 (Ti around 0.9) was employed with MACCS fingerprints. All clusters containing at least five compounds were considered for selectivity analyses. For each retained cluster, the ligands therein included were classified into three different categories on the basis of their selectivity index: (a) L_on_, ligands with SI > 5 and thus selective for the on-target (hCA IX or hCA XII); (b) L_off_, ligands with SI < 0.2 and thus selective for the off-target (hCA II); (c) L_ns_, ligands with 0.2 < SI < 5 and thus non-selective. Based on this classification, the selectivity score (S_score_) of each cluster was calculated as follows:

$$S_{\mathrm{score}}\;=\;100\;\frac{L_{\mathrm{on}}\;-\;L_{\mathrm{off}}}{L_{\mathrm{on}}+L_{\mathrm{off}}+L_{\mathrm{ns}}}$$

Therefore, clusters with positive S_score_ values would predominantly include ligands with selectivity for the on-target, while negative S_score_ values indicate clusters mainly populated by compounds with selectivity for the off-target. In the double selectivity analysis, two different S_score_ values relative to hCA II/IX and hCA II/XII selectivity were calculated for each cluster of ligands.

### KNIME workflow implementation

A KNIME workflow^22^ allowing to perform the whole selectivity profile analysis in few clicks was implemented. The workflow comprised three input nodes, a central meta node performing all calculations required for the analysis and three output nodes for visualizing the results. A *File Reader* node was used to import the full list of hCA ligands previously generated, while two *String Input* nodes were employed to select the on-target and the off-target to be considered in the analysis. Within the meta node, two initial *Row Filter* nodes were used to select from the full list of hCA ligands only those showing *K*_i_ values for the desired hCA isoforms, thus obtaining the proper dataset of compounds to be analyzed. An *RDKit Fingerprint* node was used to generate MACCS fingerprints for the dataset ligands, which were employed for the calculation of the ligand similarity matrices. Ti and Td values for all possible fingerprint pairs were calculated using a *Distance Matrix* node applying the same formula reported above, while the hierarchical clustering analysis was performed through two different KNIME nodes: a first one generating the clusters and a second one assigning the cluster labels to the different dataset ligands. The same clustering algorithm and parameters employed in orange-canvas were also used in KNIME. The S_score_ values relative to the generated clusters were obtained through a KNIME node encoding our in-house python scripts that calculate the SI value associated to each ligand and the Score associated to each cluster, based on the above reported equation. A further *Row Filter* node was then used to select the clusters with S_score_ > 70. The final nodes added at the end of the workflow produced two different visual outputs: (a) a *Render to Image* node and an *Excel Writer* node were used to generate a spreadsheet including 2D structures, CID codes and SI values of the ligands included in clusters with selectivity for the on-target (S_score_ > 70), together with the number of their corresponding cluster; (b) a *Marvin View* node was also employed to produce a compound table showing the ligands grouped within the selective clusters as visualized in Instant JChem software, which included the 2D structures of the ligands and the same properties reported in the spreadsheet.

## Results and discussion

As a first step in the development of our selectivity analysis platform, we focused on gathering a large amount of bioactivity data related to hCAs inhibition and the corresponding structures of small-molecule ligands experimentally tested for hCAs inhibitory activity. For this purpose, we searched the PubChem database and retrieved the SMILES strings of all compounds for which the result of a biological assay on at least a single hCA isoform was stored. In this way, we created 13 initial targeted datasets of ligands, each including their structure and bioactivity information related to a specific enzyme out of the 13 hCA isoforms that were considered in our analysis: hCA I–IV, Va, Vb, VI, VII, IX and XI–XIV. These preliminary data sets were then refined in order to obtain ensembles of compounds with bioactivity data that could be safely compared to each other, thus limiting the biases associated to experimental procedures. For this reason, only compounds whose inhibitory activity for the corresponding hCA isoform was expressed by a *K*_i_ value were retained in the refined datasets, while ligands associated with activity values expressed through different metrics were filtered out (see Materials and Methods for details). The 13 final datasets of CAIs were eventually merged into a single database to be used for selectivity profile analyses, including all compounds presenting a *K*_i_ value for at least one out of the 13 hCA isoforms considered in this study. However, since the majority of CAIs are characterized by the presence of a sulfonamide or sulfonamide-like moiety acting as zinc-binding group (ZBG), and the most potent CAIs belong to this class of compounds^23^, we decided to develop and test our platform for selectivity analyses considering only ligands presenting a sulfonamide fragment, which were selected through a substructure search by Instant JChem software. In this way, we obtained a final database of 2837 ligands bearing sulfonamide fragments and *K*_i_ values for one or more hCA isoforms. Table 1 shows the number of bioactivity data available for each of the 13 hCA isoforms considered in this study, relative to the ligands included in the final database employed for selectivity analyses.

**Table 1.** Bioactivities for the different hCA isoforms relative to the final database ligands.

Target isoform	Bioactivity data (*K*_i_)
hCA I	1958
hCA II	2597
hCA III	32
hCA IV	204
hCA Va	171
hCA Vb	130
hCA VI	106
hCA VII	306
hCA IX	1941
hCA XI	7
hCA XII	1531
hCA XII	42
hCA XIV	348

As a second step in our study, we wanted to assess the possibility of identifying a correlation between the structural similarities of CAIs and their selectivity for a specific hCA isoform over others in an automated way, allowing the development of a protocol exclusively based on ligands’ structures, without taking into account any kind of information concerning the structure of the target proteins. Due to the recently emerged interest in hCA IX as a new potential target for the treatment of hypoxic tumors, we decided to use hCA IX as a test target for this analysis, while considering the ubiquitously expressed hCA II as the corresponding off-target over which the selectivity of compounds would have been desirable. Therefore, we selected the 1756 ligands showing *K*_i_ values for both hCA IX and hCA II, and we calculated their selectivity index (SI), defined as the ratio between their *K*_i_ values for hCA II and hCA IX. Subsequently, the structures of these ligands were translated into fingerprints, which were then compared to one another by calculating the Tanimoto similarity index (Ti). In this way, it was possible to perform a rapid evaluation of the reciprocal structural similarity among all 1756 ligands belonging to this subset (see Materials and Methods for details). Such analysis was performed four times, using four different fingerprint types: FP2, FP3, FP4 and MACCS. Each of these fingerprints encoded the structures of the dataset ligands in a different way and thus different similarities and corresponding Ti values were obtained for the same ligand pairs. For all possible pairs of ligands included in the dataset, the Ti values of the corresponding fingerprints were calculated, together with the selectivity ratio (SR), defined as the ratio between the SI of two ligands expressed in logarithmic scale. In this way, we could evaluate the correlation between ligand similarity in terms of structure (described by the Ti) and isoform selectivity (described by the SR). A pair of ligands with a similar selectivity profile (and thus similar SI values) will show an SR close to zero, while pairs of ligands with different selectivity profiles (and thus different SI values) will show an SR far from zero. The results of each similarity analysis were evaluated by plotting Ti versus SR values calculated for each ligand pair within the dataset.

**Figure 1.**
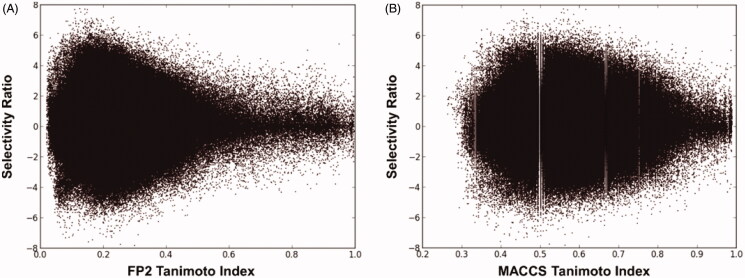
Graphical correlation between the Ti and SR of the possible dataset ligand pairs, using FP2 fingerprints (A) and MACCS fingerprints (B).

As shown in Figure 1(A), when FP2 fingerprints were used to describe ligand structures, a considerable correlation between structural similarity and selectivity profile emerged. In fact, ligands with low structural similarity (0 < Ti < 0.4) displayed very different selectivity profiles for hCA IX and II, showing SR values ranging from −8 to +8 (corresponding to differences in SI values up to eight logarithmic units). On the contrary, along with the increase of structural similarity we observed a corresponding reduction of the SR range. In particular, most of ligand pairs with high structural similarity (0.8 < Ti < 1) showed SR values close to zero, which ranged from −2 to +2, thus suggesting that similar ligands (measured with the FP2 fingerprints) show similar selectivity. A comparable although less marked correlation between ligand structural and selectivity similarity was obtained when MACCS fingerprints were used (Figure 1(B)). Differently, a poor and completely absent correlation between Ti and SR values for the dataset ligand pairs were obtained when compound structures where described by FP4 and FP3 fingerprints, respectively (Supplementary Figure S1).

**Figure 2.**
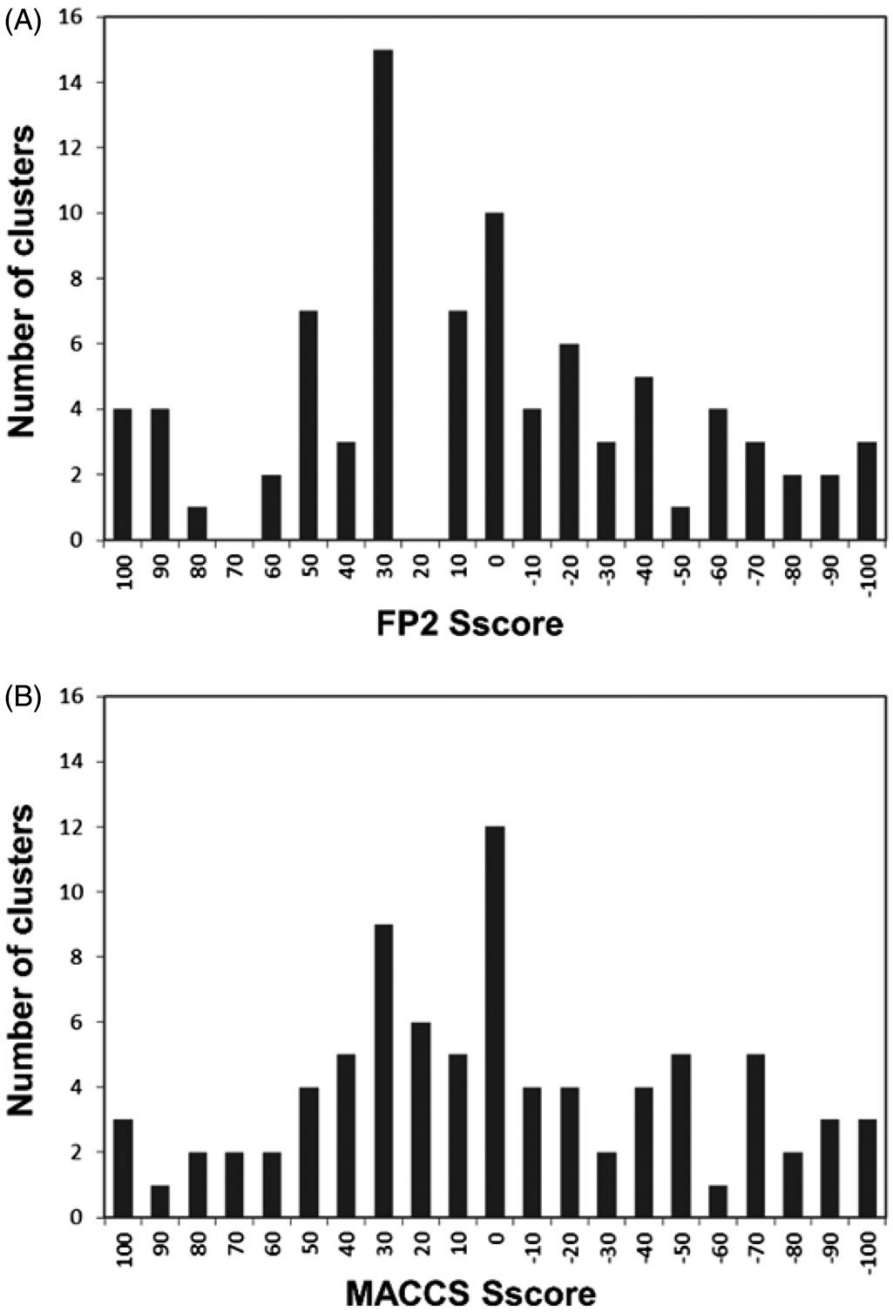
Classification analysis of the different clusters of dataset ligands generated using FP2 (A) and MACCS (B) fingerprints.

This analysis highlighted a correlation between the selectivity profile of hCA ligands and their structural features, suggesting the possibility to identify common structural motifs in hCA ligands that could be associated with their selectivity for a desired hCA isoform, thus providing useful guidelines for the design of selective CAIs. For this purpose, we clustered the dataset ligands using orange-canvas software, which performs various methods of hierarchical clustering based on distance metrics. In this case, we performed a single-linkage clustering of the dataset ligands based on their structural similarity described as Tanimoto distances. This analysis was performed considering the Ti values calculated using both FP2 and MACCS fingerprints; for this reason, we generated all possible clusters of ligands with high reciprocal Ti values according to both fingerprint types (see Materials and Methods for details). The generated clusters were then analyzed based on the selectivity of the ligands included therein. Initially, the compounds were divided into three different classes based on their SI: ligands selective for the on-target hCA IX (SI > 5) were defined as L_on_, ligands selective for the off-target hCA II (SI < 0.2) were defined as L_off_, while non-selective ligands (0.2 < SI < 5) were defined as L_ns_. Subsequently, a selectivity score (S_score_) ranging from +100 to −100 was calculated for each cluster based on the distribution of L_on_, L_off_, and L_ns_ compounds therein included (see Materials and Methods for details). An S_score_ value of +100 would be obtained for a cluster populated only by L_on_ ligands, while clusters including only L_off_ ligands would show an S_score_ value of −100. Clusters containing L_on_ and L_off_ ligands in similar number and/or including a high number of L_ns_ compounds would show S_score_ values close to zero. Figure 2 shows the distribution of the Score values calculated for all the different clusters generated for the dataset ligands using FP2 and MACCS fingerprints.

**Figure 3.**
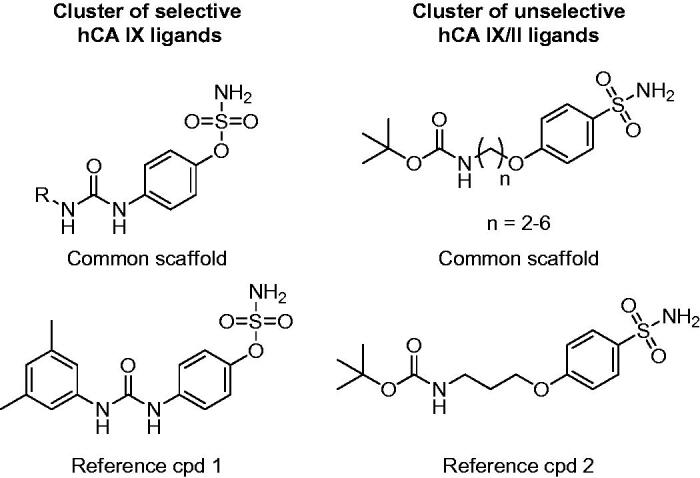
Representative clusters of selective hCA IX and unselective hCA IX/II ligands. The common scaffolds identified in these clusters are shown, together with a reference compound.

Both procedures allowed the classification of the dataset ligands into many different clusters with rather homogenously distributed values of S_score_. Notably, in both cases a considerable number of selective clusters, counting up to 41 compounds and predominantly including either L_on_ or L_off_ ligands, were generated. By visually inspecting the compounds populating these clusters it was possible to identify structural elements associated with hCA IX selectivity. Figure 3 shows the common core structure identified in the most populated highly selective cluster (S_score_ = +100) of hCA IX ligands identified using MACCS fingerprints. All 41 ligands included in this cluster share a sulfamate moiety as ZBG and present a ureidic fragment connected to it through a phenyl ring. All these compounds are endowed with high inhibitory activity against hCA IX, with *K*_i_ values ranging from 5 to 100 nM, and an 8 to 80-fold selectivity for hCA IX over hCA II. Compound 1 (Figure 3) represents one of the most potent and selective ligands of this cluster (*K*_i_ for hCA IX = 7 nM, SI = 78). Therefore, it appears that this particular structural motif would be able to confer the desired selectivity profile to new potential CAIs designed based on the same scaffold. Our selectivity profile analysis was also suitable for identifying structural motifs shared by promiscuous ligands, which should be thus avoided in the design of selective CAIs and taken into account only for the design of ligands targeting multiple hCA isoforms (in this case hCA IX and II). A remarkable example of a completely non-selective cluster (S_score_ = 0) is represented by a small set of ligands with a well-defined structure, bearing a phenylsulfonamide head and an N-tert-butoxycarbonyl tail connected by an alkoxy linker. The compounds belonging to this cluster show double-digit nanomolar *K*_i_ values for both hCA IX and II and completely lack of any selectivity. For instance, compound 2 (Figure 3) shows *K*_i_ values of 58 and 51 nM for hCA IX and hCA II, respectively.

**Figure 4.**
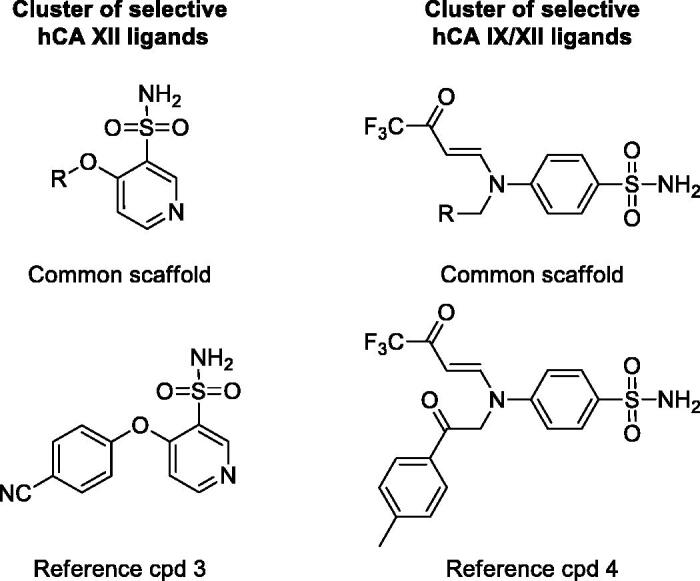
Representative clusters of selective hCA XII and double-selective hCA IX-XII ligands. The common scaffolds identified in these clusters are shown, together with a reference compound.

As a further evaluation of our selectivity profiling platform, we repeated the whole analysis considering hCA XII as the on-target and maintaining hCA II as the off-target. For this purpose, we selected a dataset of 1388 ligands showing *K*_i_ values for both hCA XII and hCA II, we calculated the SI for each compound and proceeded performing the structural similarity analysis. In this case, only FP2 and MACCS fingerprints were used to describe the structures of the dataset ligands and calculate their reciprocal similarity. The compounds were then partitioned into different clusters based on the Ti values calculated for all possible ligand pairs within the dataset, as previously performed, and the so obtained clusters were again analyzed calculating the corresponding S_score_. The analysis revealed again the presence of highly selective clusters with an S_score_ of +100 (Supplementary Figure S2), thus completely populated by L_on_ ligands (selective for hCA XII over hCA II). Interestingly, the sulfamate inhibitors included in the above mentioned cluster of hCA IX selective compounds (Figure 3) were also found to be particularly selective for hCA XII with respect to hCA II. In fact, most of these ligands showed single-digit *K*_i_ values for hCA XII and a range of SI reaching values above 100. For instance, compound 1 was found to be the most selective ligand of the cluster, with SI = 273 and a *K*_i_ value for hCA XII inhibition of 2 nM. Another cluster of single-digit nanomolar inhibitors with good selectivity for hCA XII is shown in Figure 4. Despite their low molecular weight, these ligands present *K*_i_ values for hCA XII inhibition around 6 nM and SI values around 13.5, as observed for compound 3 (*K*_i_ = 6.3 nM, SI = 15).

Based on these results, we thought about the possibility of applying the same procedure to identify ligands selective for more than one hCA isoform at the same time. For this reason, we selected the 1322 compounds showing *K*_i_ values for both hCA II, IX and XII, and repeated the whole analysis. In this case, only MACCS fingerprints were used, since they demonstrated to perform better than FP2 for clustering purposes in the previous analyses, generating clusters of ligands with a more defined central scaffold shared by all compounds. Moreover, for each generated cluster of compounds, we separately calculated two different S_score_ values relative to hCA II/IX and hCA II/XII selectivity. The cluster evaluation was then performed based on the analysis of the two S_score_ values. The most interesting clusters were visually inspected in order to identify common structural motifs already highlighted in the previous analysis. As expected, the sulfamate ligands represented by compound 1 (Figure 3) were correctly identified as double-selective hCA IX/XII compounds and grouped in a single cluster that showed a double S_score_ (the sum of the two different S_score_ values) of +200. Moreover, the analysis identified another small cluster of compounds with high double S_score_, endowed with remarkable activity and selectivity for both hCA IX and XII; these compounds are characterized by the presence of a trifluoroacetyl group and a Y-shaped structure (Figure 4), as observed in compound 4 (*K*_i_ for hCA IX and XII of 18 nM and 28 nM, respectively; SI for hCA IX and XII of 22.7 and 14.6, respectively). Conversely, the cluster of ligands represented by compound 2 (Figure 2), was found to be totally unselective also for hCA XII (double S_score_ = 0); therefore, these molecules were confirmed to be promiscuous CAIs. The results of these double selectivity analysis demonstrated the reliability of our protocol in identifying ligands with selectivity for multiple hCA isoforms at the same time.

**Figure 5.**
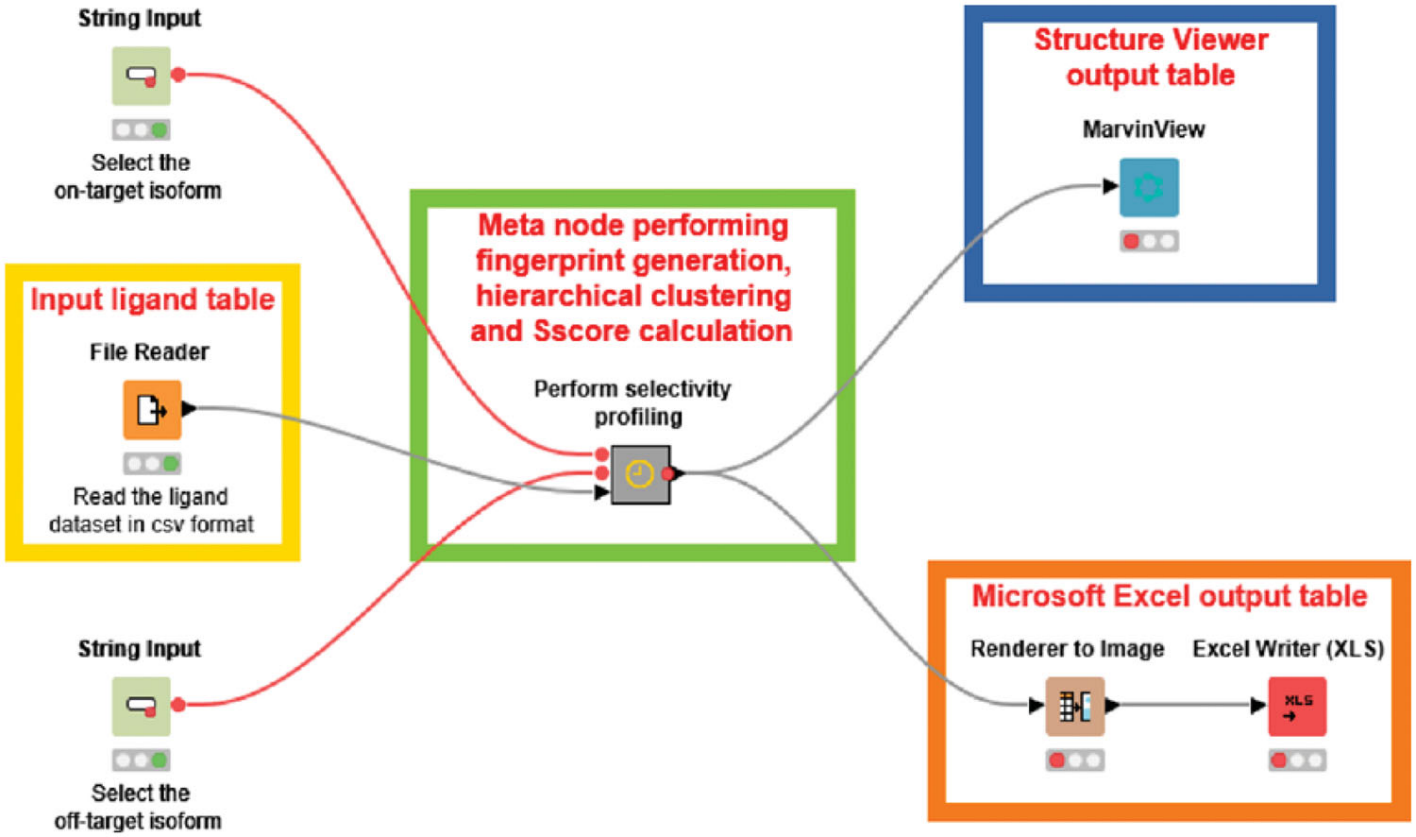
KNIME workflow for automatic selectivity profile analyses.

With the aim of automatizing our protocol for selectivity profile analyses and make it accessible even to the non-expert user, we developed a KNIME Analytic Platform workflow combining all the different steps included in the protocol. The developed workflow, shown in Figure 5, allows to perform in just few clicks a complete selectivity analysis such as those herein reported as test cases for the identification of selective hCA IX or hCA XII ligands. The only input data that need to be provided by the user is the full list of CAIs containing the ligands’ identification codes (CIDs), SMILES strings and *K*_i_ values for the different hCA isoforms, as previously employed, which should be selected from the *File Reader* node. Subsequently, it is enough to set the two *String Input* nodes, specifying the on-target and off-target that should be considered for the selectivity profile analysis, and to execute the workflow. All operations required for the analysis, including fingerprint generation, hierarchical clustering and calculation of S_score_ for the obtained clusters, are sequentially performed through multiple KNIME nodes, grouped into a central meta node, as soon as the workflow is started (see Materials and Methods for details). The results of the analysis can be checked through two different output tables that are automatically produced at the end of all calculations: a Microsoft Excel spreadsheet and a MarvinView table that allows to visualize the results as in Instant JChem software, both including 2D structures, identification codes and bioactivity information for the ligands belonging to clusters with S_score_ >70, thus selective for the on-target.

## Conclusions

The selectivity for a desired hCA isoform is a pivotal feature that must be taken into account when developing novel hCA ligands with the aim of obtaining valuable therapeutic tools for the treatment of a desired pathology, with low probability of showing adverse effects due to residual activity against off-target CAs. In the present study, we developed an efficient protocol that allows to analyze the selectivity profile of the different CAIs reported in literature. By using in-house python scripts, we were able to obtain a comprehensive dataset of CAIs including ligand structures and bioactivity data for the different hCA isoforms, which were retrieved from the publicly accessible PubChem database. The dataset was then used to develop and validate a chemoinformatic platform for selectivity profile analyses exclusively based on the comparison of ligand fingerprints and bioactivity data, thus requiring neither information about the structure of the target proteins nor about the binding mode of the analyzed ligands. Our platform allows an ultrafast ligand-based analysis that highlights the presence of structure-selectivity relationships, i.e. the identification of common structural motifs shared by ligands endowed with a similar selectivity profile. In the context of the case studies herein reported, the platform was able to identify specific clusters of ligands with high activity and selectivity for either hCA IX or hCA XII over hCA II, as well as compounds with double selectivity for hCA IX and XII over hCA II, thus providing useful guidelines for the design of selective inhibitors of the tumor-related hCA isoforms. At the same time, the platform highlighted the presence of completely non-selective clusters of compounds with similar activities against the three hCA isoforms, thus also demonstrating its efficacy in identifying useful structural motifs for the potential design of pan-selective compounds. The developed platform was implemented as a KNIME workflow, thus allowing an ultrafast selectivity profile analysis that can be performed in just few clicks and be even accessible to non-expert users. The platform is applicable for selectivity profile analyses focused on all types of ligands and all kinds of protein targets for which a considerable number of bioactive compounds is available in PubChem database. The platform will be made available to all users as a toolbox on our research group website: http://www.mmvsl.it.

## Supplementary Material

Supplemental Material
