# Supplementary material for: Development of a cheminformatics platform for selectivity analyses of carbonic anhydrase inhibitors
Source: J Enzyme Inhib Med Chem. 2019 Dec 19;35(1):365–71. doi: 10.1080/14756366.2019.1705291 (PMC6968703; doi:10.1080/14756366.2019.1705291)
Supplement: Supplemental Material [file IENZ_A_1705291_SM4416.pdf]

Supporting Information

Development of a cheminformatics platform for selectivity analyses of carbonic anhydrase inhibitors.

Giulio Poli,[1] Salvatore Galati,[1] Adriano Martinelli,[1] Claudiu T Supuran,[2] Tiziano Tuccinardi[1]

[1]Department of Pharmacy, University of Pisa, Pisa, Italy, [2]NEUROFARBA Department, Sezione di Scienze Farmaceutiche, Università degli Studi di Firenze, Sesto Fiorentino, Florence, Italy.

Address for correspondence: Tiziano Tuccinardi, Department of Pharmacy, University of Pisa, Via Bonanno 6, 56126 Pisa, Italy. E-mail: tiziano.tuccinardi@unipi.it

Table of Content

Figure S1. Correlation between the Ti and SR using FP3 and FP4 fingerprints. Pag. S1

Figure S2. Cluster classification analysis for hCA II/XII selectivity. Pag. S1

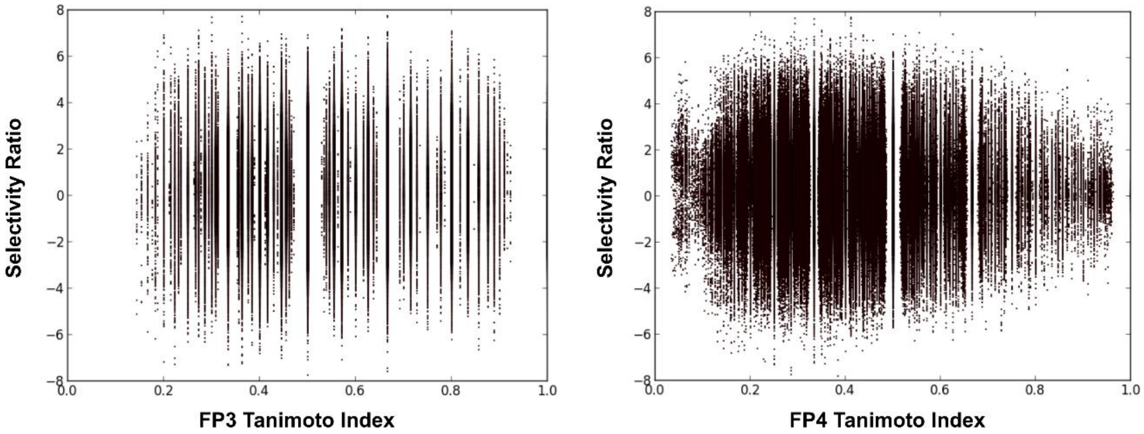

Figure S1. Graphical correlation between the Ti and SR of the possible dataset ligand pairs, using FP3 fingerprints (left) and FP4 fingerprints (right).

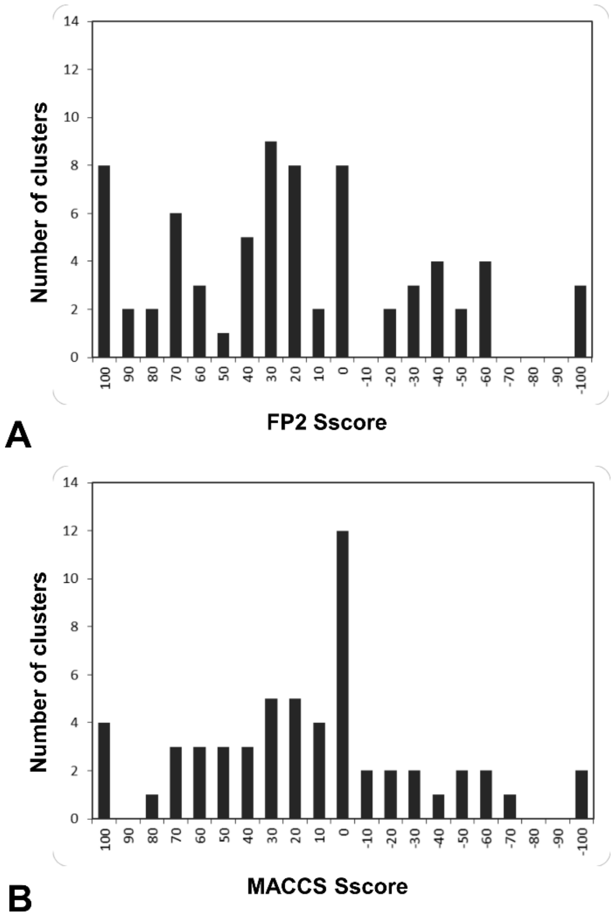

Figure S2. Classification analysis of the different clusters of dataset ligands generated using FP2 (A) and MACCS (B) fingerprints.
